# Supplementary material for: Occupation and Sickness Absence in the Different Autonomous Communities of Spain
Source: Int J Environ Res Public Health. 2021 Oct 30;18(21):11453. doi: 10.3390/ijerph182111453 (PMC8583026; doi:10.3390/ijerph182111453)
Supplement: Supplementary file 1 [file ijerph-18-11453-s001.zip › ijerph-1376558-supplementary.pdf]

Table S1: Descriptive analysis by ISCO-08 sickness absence duration in the Autonomous Communities in Spain

Managers		*Professionals*		*Technicians and associate professionals*	
**Average**	69,17	**Average**	41,96	**Average**	55,57
**Standard error**	4,98	**Standard error**	2,11	**Standard error**	3,28
**Median**	74,19	**Median**	43,24	**Median**	58,05
**Standard deviation**	21,69	**Standard deviation**	9,19	**Standard deviation**	14,31
**Variance**	470,37	**Variance**	84,43	**Variance**	204,75
**Kurtosis**	-1,13	**Kurtosis**	-0,81	**Kurtosis**	-1,34
**Skewness**	-0,29	**Skewness**	-0,18	**Skewness**	-0,16
**Range**	66,69	**Range**	30,02	**Range**	43,59
**Minimun**	33,56	**Minimun**	25,44	**Minimun**	33,64
**Maximun**	100,26	**Maximun**	55,46	**Maximun**	77,23
**Sum**	1314,22	**Sum**	797,30	**Sum**	1055,76

Clerical support workers		*Service and sales workers*		*Skilled agricultural, forestry and fishery workers*	
**Average**	48,76	**Average**	52,17	**Average**	50,47
**Standard error**	2,58	**Standard error**	2,91	**Standard error**	2,74
**Median**	48,43	**Median**	53,24	**Median**	50,84
**Standard deviation**	11,24	**Standard deviation**	12,69	**Standard deviation**	11,94
**Variance**	126,43	**Variance**	161,05	**Variance**	142,60
**Kurtosis**	-1,16	**Kurtosis**	-1,29	**Kurtosis**	-1,24
**Skewness**	-0,11	**Skewness**	-0,13	**Skewness**	-0,12
**Range**	35,41	**Range**	39,28	**Range**	37,34
**Minimun**	30,43	**Minimun**	32,25	**Minimun**	31,34
**Maximun**	65,84	**Maximun**	71,54	**Maximun**	68,69
**Sum**	926,53	**Sum**	991,14	**Sum**	958,84

Craft and related trades workers		*Plant and machine operators, and assemblers*		*Elementary occuations*	
**Average**	51,32	**Average**	50,89	**Average**	51,10
**Standard error**	2,82	**Standard error**	2,78	**Standard error**	2,80
**Median**	52,04	**Median**	51,44	**Median**	51,74
**Standard deviation**	12,31	**Standard deviation**	12,12	**Standard deviation**	12,22
**Variance**	151,54	**Variance**	147,00	**Variance**	149,25
**Kurtosis**	-1,27	**Kurtosis**	-1,26	**Kurtosis**	-1,26
**Skewness**	-0,12	**Skewness**	-0,12	**Skewness**	-0,12
**Range**	38,31	**Range**	37,83	**Range**	38,07
**Minimun**	31,80	**Minimun**	31,57	**Minimun**	31,68

**Maximun**	70,11	**Maximun**	69,40	**Maximun**	69,75
**Sum**	974,99	**Sum**	966,91	**Sum**	970,95

Table S2. Number of sickness absences cases by CIE-10 and ISCO-08 (I).

	Uninformed	Managers	Professionals	Technicians and associate professionals	Clerical support workers	Service and sales workers	Skilled agricultural, forestry and fishery workers	Craft and related trades workers	Plant and machine operators and assemblers	Elementary occupations
**Certain infectious and parasitic diseases**	138672	27	288	151	374	633	43	126	182	488
**Neoplasms**	65427	671	2909	1802	2762	4807	537	1288	1397	3950
**Diseases of the blood and blood-forming organs and certain disorders involving the immune mechanism**	5395	20	114	58	93	197	27	42	49	183
**Endocrine, nutritional and metabolic diseases**	14943	35	238	126	242	563	53	145	202	466
**Mental and behavioural disorders**	212245	1166	6813	3566	6792	12741	773	2206	3046	7467
**Diseases of the nervous system**	56704	246	1036	740	1177	2763	386	887	1211	2854
**Diseases of the eye and adnexa**	67997	101	544	312	630	868	143	299	430	783
**Diseases of the ear and mastoid process**	43633	37	268	154	290	517	57	155	255	520

Table S2. Number of sickness absences cases by CIE-10 and ISCO-08 (II).

	Uninformed	Managers	Professionals	Technicians and associate professionals	Clerical support workers	Service and sales workers	Skilled agricultural, forestry and fishery workers	Craft and related trades workers	Plant and machine operators and assemblers	Elementary occupations
**Diseases of the circulatory system**	65314	465	1183	1075	1255	3257	586	1388	1946	3004
**Diseases of the respiratory system**	399925	112	820	432	973	1579	173	457	580	1626
**Diseases of the digestive system**	184194	227	883	687	1029	2449	330	819	959	2355
**Diseases of the skin and subcutaneous tissue**	49506	39	162	162	218	610	88	231	255	594
**Diseases of the musculoskeletal system and connective tissue**	751367	1676	6527	5704	7804	26448	4567	6683	9267	28626
**Diseases of the genitourinary system**	107819	117	544	356	738	1619	167	397	457	1541
**Pregnancy, childbirth and the puerperium**	58871	16	524	121	187	452	18	66	23	315

Table S2. Number of sickness absences cases by CIE-10 and ISCO-08 (III).

	Uninformed	Managers	Professionals	Technicians and associate professionals	Clerical support workers	Service and sales workers	Skilled agricultural, forestry and fishery workers	Craft and related trades workers	Plant and machine operators and assemblers	Elementary occupations
**Congenital malformations, deformations and chromosomal abnormalities**	6238	10	80	56	98	239	25	74	80	227
**Symptoms, signs and abnormal clinical and laboratory findings, not elsewhere classified**	224899	246	1249	910	1783	3108	274	854	1175	2977
**Injury, poisoning and certain other consequences of external causes**	292232	563	3097	1905	2416	6936	973	2438	2616	5976
**External causes of morbidity and mortality**	757	2	6	2	7	15	5	11	5	11
**Factors influencing health status and contact with health services**	14416	17	114	46	89	203	29	61	60	173
**Codes for special purposes**	308724	616	2551	1931	3211	7998	999	2003	2254	7204
